# Effect of a multispecies probiotic supplement on quantity of irritable bowel syndrome-related intestinal microbial phylotypes

**DOI:** 10.1186/1471-230X-10-110

**Published:** 2010-09-19

**Authors:** Anna Lyra, Lotta Krogius-Kurikka, Janne Nikkilä, Erja Malinen, Kajsa Kajander, Kyösti Kurikka, Riitta Korpela, Airi Palva

**Affiliations:** 1 Department of Veterinary Biosciences, Faculty of Veterinary Medicine, University of Helsinki, Helsinki, Finland; 2 Current Address: Danisco Sweeteners, Health and Nutrition, Kantvik, Finland; 3 Valio Ltd., Research Centre, Helsinki, Finland; 4 Current Address: Oy Verman Ab, Kerava, Finland; 5 Numos Ltd., Espoo, Finland; 6 Institute of Biomedicine, University of Helsinki, Helsinki, Finland

## Abstract

**Background:**

Probiotics can alleviate the symptoms of irritable bowel syndrome (IBS), possibly by stabilizing the intestinal microbiota. Our aim was to determine whether IBS-associated bacterial alterations were reduced during multispecies probiotic intervention consisting of *Lactobacillus rhamnosus *GG, *L. rhamnosus *Lc705, *Propionibacterium freudenreichii *ssp. *shermanii *JS and *Bifidobacterium breve *Bb99. The intervention has previously been shown to successfully alleviate gastrointestinal symptoms of IBS.

**Methods:**

The faecal microbiotas of 42 IBS subjects participating in a placebo-controlled double-blind multispecies probiotic intervention were analysed using quantitative real-time polymerase chain reaction (qPCR). Eight bacterial targets within the gastrointestinal microbiota with a putative IBS association were measured.

**Results:**

A phylotype with 94% similarity to *Ruminococcus torques *remained abundant in the placebo group, but was decreased in the probiotic group during the intervention (P = 0.02 at 6 months). In addition, the clostridial phylotype, *Clostridium thermosuccinogenes *85%, was stably elevated during the intervention (P = 0.00 and P = 0.02 at 3 and 6 months, respectively). The bacterial alterations detected were in accordance with previously discovered alleviation of symptoms.

**Conclusions:**

The probiotic supplement was thus shown to exert specific alterations in the IBS-associated microbiota towards the bacterial 16S rDNA phylotype quantities described previously for subjects free of IBS. These changes may have value as non-invasive biomarkers in probiotic intervention studies.

## Background

Irritable bowel syndrome (IBS), a common functional gastrointestinal (GI) disorder, is characterized by abdominal pain or discomfort, diarrhoea, constipation, abdominal bloating and flatulence, which are associated with changes in the frequency and form of stool and may markedly lower the quality of life [1]. The diagnosis of IBS is still symptom-based, emphasizing the need for non-invasive biomarkers in diagnosis and therapeutic trial follow-up [2]. Multiple features affect IBS aetiology, including stress, altered GI motility and visceral hypersensitivity [3,4]. In addition, abundant evidence suggests microbial involvement in IBS. Low-grade mucosal inflammation has been observed in the GI tract of IBS patients, whereas onset of GI symptoms after gastroenteritis generates a subset of patients diagnosed with post-infectious IBS [5,6]. Several observations have suggested the presence of an altered GI microbiota among IBS subjects [7-13] and that probiotics may alleviate IBS symptoms [14,15] with several mechanisms of action [16].

The bacterial species *Lactobacillus *spp., *Veillonella *spp. and *Bifidobacterium *spp. and the groups *Clostridium coccoides *and *Bifidobacterium catenulatum *are affected in IBS [8]. In addition, alterations in the abundance of several 16S rRNA gene phylotypes have been observed [9,11]. These include phylotypes from the families *Lachnospiraceae, Ruminococcaceae, Erysipelotrichaceae, Bacteroidaceae, Coriobacteriaceae *and a novel *Firmicutes *phylotype with 85% similarity to *Clostridium thermosuccinogenes*. However, in quantitative real-time polymerase chain reaction (qPCR) analyses [8,9,11], the overall microbiota is not covered, as the quantified bacteria are predetermined according to primer sequences. With a phylogenetic microarray covering over 1000 human faecal phylotypes, the GI microbiota of IBS patients was shown to diverge from that of healthy controls, with comparably strong variation seen among IBS patients [13]. Furtherrmore, in a 16S rDNA clone library sequencing study, the GI microbiota of diarrhoea-predominant IBS (IBS-D) subjects had relatively high numbers of *Proteobacteria *and *Firmicutes *(especially family *Lachnospiracheae*) and low numbers of *Actinobacteria *and *Bacteroidetes *[12].

A multispecies probiotic combination (*Lactobacillus rhamnosus *GG, *L. rhamnosus *Lc705, *Propionibacterium freudenreichii *ssp. *shermanii *JS and *Bifidobacterium breve *Bb99), which was assessed in this study, was earlier found to significantly alleviate IBS symptoms in a 6-month placebo-controlled intervention [17]. The total symptom score of IBS patients ingesting the probiotic combination was significantly lowered due to less borborygmi [17]. Alterations in the GI microbiota were later monitored by quantitative real-time polymerase chain reaction (qPCR) and analysis of short-chain fatty acid content and bacterial enzyme levels [18], but microbial factors were concluded not to be responsible for the observed effect. However, we continued the analyses of the original intervention samples since novel 16S rRNA gene phylotype targeting assays were later shown to differentiate between IBS patients and healthy control subjects devoid of GI symptoms [11].

Here, we present the analysis of intervention samples with eight 16S rRNA phylotype-targeting qPCR assays. The multispecies probiotic supplement shifts the intestinal microbiota of IBS subjects towards that associated with healthy control subjects.

## Methods

### Study design and subjects

The 6-month probiotic intervention study was originally conducted as a randomized, double-blind, placebo-controlled intervention [17]. IBS patients received daily either a probiotic capsule (Valio Ltd., Helsinki, Finland) containing *L. rhamnosus *GG (ATCC 53103, LGG), *L. rhamnosus *Lc705 (DSM 7061, Lc705), *P. freudenreichii *ssp. *shermanii *JS (DSM 7067, PJS) and *B. breve *Bb99 (DSM 13692, Bb99) or a placebo capsule consisting of microcrystalline cellulose, magnesium stearate and gelatine as an encapsulating material. The total daily amount of bacteria in the probiotic capsule was 8-9 × 10^9 ^colony forming units, with an equal amount of each strain. Consumption of other probiotic products was not allowed during the intervention. All subjects were advised to follow their usual dietary habits and to not make any changes to their medication, including ongoing IBS medication (mainly commercial fibre analogues, laxatives or antidiarrhoeals).

Participants fulfilled the Rome II criteria [19], except for three subjects who reported slightly less than 12 weeks of abdominal pain during the preceding year. All patients had undergone a clinical investigation and endoscopy or barium enema of the GI tract 0-1 year prior to the study. Exclusion criteria for participation were pregnancy, lactation, organic intestinal disease, other severe systematic disease, antimicrobial medication during the preceding two months, previous major or complicated abdominal surgery, severe endometriosis and dementia or otherwise inadequate cooperation capability. Patients with lactose intolerance were allowed to participate if they reported following a low-lactose or lactose-free diet. A total of 22 IBS patients receiving a multispecies probiotic and 20 IBS patients receiving a placebo capsule were analysed at the time-points of 0, 3 and 6 months (Table 1). The faecal samples of the placebo group [7-9,11,12,17,20] and of both the placebo and probiotic groups [18], had been studied previously with different approaches.

**Table 1:** Characteristics of irritable bowel syndrome subjects (n = 42).

	Multispecies probiotic	Placebo
Age (years): mean (range)	46 (28-63)	47 (24-64)
Gender: F/M	15/7	14/6
Predominant bowel habit		
Diarrhoea: n	11	8
Constipation: n	3	8
Alternating: n	8	4

### Ethics

All patients gave their written informed consent and were told that they could withdraw from the study at any time. The Human Ethics Committee of the Joint Authority for the Hospital District of Helsinki and Uusimaa (HUS) approved the study protocol.

### Extraction and purification of DNA from faecal samples

Faecal samples were stored anaerobically immediately after defecation, then mixed and aliquoted and finally stored at -70°C within 4 h of delivery. Bacterial DNA was isolated from 1 g of faecal material by removing the undigested particles from the faecal mass using three rounds of low-speed centrifugation, collection of bacterial cells with high-speed centrifugation, enzymatic and mechanical cell lysis and DNA extraction and precipitation [21]. A NanoDrop ND-1000 Spectrophotometer (NanoDrop Technologies, Wilmington, DE, USA) was used to determine the DNA concentrations.

### qPCR assays for quantifying faecal bacterial phylotypes

The qPCR assays targeted intestinal bacterial phylotypes associated with IBS (Table 2) [9,11]. The iCycler iQ Real-Time Detection System (Bio-Rad, Hercules, CA, USA), a component of iCycler Optical System Interface software (version 2.3; Bio-Rad), was used to analyse the samples as described previously [9,11]. Standards ranged from 10^2 ^to 10^7 ^16S rRNA gene copies per reaction.

**Table 2:** qPCR primers and assay conditions.

Assay	Primers (5' → 3')	Standard	Classification of standard ( > 98% unless otherwise stated)	Target size (bp)	MgCl_2_ (mM)	Annealing T (°C)	Detection T (°C)
*Bacteroides intestinalis*-like [11]	F: AGCATGACCTAGCAATAGGTT R: CCTTCTCGTTATACTATCCGGTAT	[EMBL:AM277809]	*Bacteroides*	124	3	63	83
*Clostridium cocleatum *88% [9]	F: AATACATAAGTAACCTGGCRTC R: CGTAGCACTTTTCATATAGAGTT	[EMBL:AM276544]	*Erysipelotrichaceae*	104	4	60	80
*Clostridium thermosuccinogenes *85% [11]	F: ACATGCAAGTCGAACGGAAGTC R: TGCGTCAGAGTTTCCTCCATTG	[EMBL:AM275406]	*Clostridiales *97%	373	2	62	81
*Collinsella aerofaciens*-like [9]	F: CCCGACGGGAGGGGAT R: CTTCTGCAGGTACAGTCTTGAC	[EMBL:AM276090]	*Collinsella*	260	4	67	89
*Coprococcus eutactus*-like [9]	F: AGCTTGCTCCGGCYGATTTA R: CGGTTTTACCAGTCGTTTCCAA	[EMBL:AM275825]	*Coprococcus*	97	2	63	83
*Ruminococcus torques *91% [9]	F: TGCTTAACTGATCTTCTTCGGA R: CGGTATTAGCAGTCATTTCTG	[EMBL:AM276624]	*Lachnospiraceae*	119	5	62	82
*Ruminococcus torques *93% [11]	F: GACTGCTTTTGAAACTGTCA R: AGGTCCGGTTAAGGA	[EMBL:AM275798]	*Lachnospiraceae*	396	4	61	83
*Ruminococcus torques *94% [9]	F: AATCTTCGGAGGAAGAGGACA R: ACACTACACCATGCGGTCCT	[EMBL:AM275522]	*Lachnospiraceae*	137	2	65	85

### Statistical analysis

Undetected abundances in the data were imputed with mean values obtained from qPCR runs with the same primer applied to water. If water runs were undetected for a certain assay, the lowest value of all detected water runs was used. All statistical analyses were conducted with log_10 _values of the number of 16S rRNA gene copies detected with the qPCR assay from the 25-ng sample of faecal DNA.

Principal component analysis (PCA) was used to get an overview of the data, and it was computed for the eight quantified bacterial phylotypes in this study and the one-week GI symptom scores (abdominal pain, distension, flatulence and borborygmi) collected in parallel with faecal samples and reported previously by Kajander *et al*. [17]. The PCA was performed separately for data collected at baseline (0 months) and during consumption of the probiotic or placebo capsule (3 and 6 months) to visualize the similarity structures in the data before the probiotic intervention and during that.

Assay-specific statistical analyses were also conducted to compare the probiotic and placebo effects, both for all IBS symptom subtypes together and for the IBS-D symptom subtype patients alone. We used standard mixed-effect linear models, with fixed effects for time and treatment and their interaction and a random intercept effect for individual (taking into account the repeated measures from the same subject). The validity of the model assumptions (homogeneity and normality of variances) was controlled by studying the residuals from the fitted models. Additionally, the need for subject-wise baseline correction was checked (no need for subject-wise baseline correction for this data). Inference from the estimated models was based on standard F-tests and t-tests. All analyses were performed with the statistical programming language R 2.6.2 [22] and utilizing the package *lme *for linear models and *contrast *for computing contrasts.

We emphasize here that despite our approach to use minimal number of tests by testing only those effects with significant effect on variance as shown by F-tests, multiple hypothesis tests are conducted and this increases the possibility that the some of the outcomes may be due chance. Partially due to this, the constipation-predominant (IBS-C) and mixed symptom subtype (IBS-M) groups were not analysed separately, because they additionally had unbalanced and small number of subjects.

## Results

### qPCR analyses

The number of 16S rDNA copies detected ranged from log_10 _± 95% confidence interval 2.02 ± 0.60 to 5.39 ± 0.17 per 25 ng of faecal DNA in the phylotype-targeting assays (Table 3). The *C. thermosuccinogenes *85%, *Ruminococcus torques *91% and *R. torques *93% phylotypes were detected in all analysed samples (Table 4). Additionally, assays targeting *Bifidobacterium catenulatum/Bifidobacterium pseudocatenulatum*-like, *Butyrivibrio crossotus*-like, *Cobrobacillus catenaformis *91% and *Slackia faecicanis *91% phylotypes [9,11] were analysed, but no alterations were detected (data not shown).

**Table 3:** Number of 16S rRNA gene copies detected.

		IBS*			IBS-D**		
qPCR assay	(m)	Probiotic (n = 22)	Placebo (n = 20)	*P*	Probiotic (n = 11)	Placebo (n = 8)	*P*
*Bacteroides intestinalis*-like	0	2.33 ± 0.69	2.65 ± 0.77	0.61	2.21 ± 0.80	1.52 ± 0.37	0.24
	3	2.49 ± 0.81	2.02 ± 0.60	0.52	2.38 ± 1.12	1.43 ± 0.60	0.20
	6	2.53 ± 0.81	2.30 ± 0.74	0.92	2.45 ± 1.14	1.19 ± 0.51	0.11
*Clostridium cocleatum *88%	0	5.17 ± 0.73	4.98 ± 0.95	0.72	5.03 ± 1.03	4.07 ± 1.66	0.23
	3	5.06 ± 0.79	5.11 ± 0.95	0.93	5.07 ± 1.03	4.52 ± 1.81	0.53
	6	5.14 ± 0.72	4.92 ± 0.85	0.72	5.39 ± 0.99	4.56 ± 1.65	0.33
*Clostridium thermosuccinogenes *85%	0	4.00 ± 0.55	3.94 ± 0.52	0.63	3.79 ± 0.63	3.52 ± 0.80	0.29
	3	5.05 ± 0.37	3.46 ± 0.39	**0.00**	4.88 ± 0.53	3.64 ± 0.47	**0.01**
	6	4.87 ± 0.41	3.91 ± 0.46	**0.02**	4.71 ± 0.67	3.54 ± 0.53	**0.05**
*Collinsella aerofaciens*-like	0	4.57 ± 0.63	4.14 ± 0.69	0.35	4.36 ± 0.96	3.13 ± 1.00	0.06
	3	4.33 ± 0.72	4.10 ± 0.71	0.70	3.91 ± 1.15	3.01 ± 0.96	0.25
	6	4.32 ± 0.68	4.45 ± 0.68	0.71	3.92 ± 1.06	3.79 ± 1.12	0.91
*Coprococcus eutactus *97%	0	2.62 ± 0.73	2.99 ± 0.69	0.84	2.95 ± 1.26	2.49 ± 0.53	0.28
	3	2.75 ± 0.65	2.94 ± 0.69	0.94	3.42 ± 1.10	2.63 ± 1.02	0.20
	6	2.77 ± 0.60	3.03 ± 0.64	0.85	3.15 ± 0.97	2.53 ± 0.78	0.22
*Ruminococcus torques *91%	0	4.29 ± 0.36	4.27 ± 0.32	0.93	4.17 ± 0.44	4.36 ± 0.47	0.60
	3	4.22 ± 0.28	4.34 ± 0.40	0.64	4.10 ± 0.42	4.54 ± 0.70	0.23
	6	4.05 ± 0.39	4.05 ± 0.40	1.00	3.73 ± 0.61	4.21 ± 0.53	0.19
*Ruminococcus torques *93%	0	5.17 ± 0.20	5.39 ± 0.17	0.19	5.04 ± 0.27	5.45 ± 0.30	0.09
	3	5.25 ± 0.17	4.42 ± 0.26	**0.00**	5.37 ± 0.28	4.70 ± 0.41	**0.00**
	6	5.00 ± 0.36	4.50 ± 0.20	**0.00**	4.73 ± 0.64	4.57 ± 0.15	0.50
*Ruminococcus torques *94%	0	3.54 ± 0.61	4.14 ± 0.53	0.10	3.69 ± 0.87	4.75 ± 0.49	**0.04**
	3	3.48 ± 0.60	3.97 ± 0.57	0.30	3.48 ± 0.92	4.42 ± 0.90	0.14
	6	3.06 ± 0.61	4.07 ± 0.43	**0.02**	2.72 ± 0.90	4.41 ± 0.66	**0.01**

* Number of 16 S rRNA gene copies detected from 25 ng of faecal DNA at each time-point analysed and the p-values for linear model comparisons of probiotic and placebo groups as a whole. The values are presented as log_10 _averages for each time-point (0, 3 and 6 months). Significant p-values (*P *< 0.5) are indicated in bold.

** Number of 16 S rRNA gene copies detected from 25 ng of faecal DNA at each time-point analysed and the p-values for linear model comparisons for diarrhoea-predominant (IBS-D) subjects. The values are presented as log_10 _averages for each time-point (0, 3 and 6 months). Significant p-values (*P *< 0.5) are indicated in bold.

**Table 4:** Prevalence of bacterial phylotypes.

qPCR assay	Placebo			Probiotic		
	**IBS-C** **(n = 8)**	**IBS-D** **(n = 8)**	**IBS-M** **(n = 4)**	**IBS-C** **(n = 3)**	**IBS-D** **(n = 11)**	**IBS-M** **(n = 8)**

*Bacteroides intestinalis*-like	7*	8	4	3	8	6
*Clostridium cocleatum *88%	7	8	4	3	11	7
*Clostridium thermosuccinogenes *85%	8	8	4	3	11	8
*Collinsella aerofaciens*-like	6	6	4	3	9	7
*Coprococcus eutactus *97%	2	3	3	1	9	5
*Ruminococcus torques *91%	8	8	4	3	11	8
*Ruminococcus torques *93%	8	8	4	3	11	8
*Ruminococcus torques *94%	8	8	3	3	10	6

* Number of subjects with target 16S rRNA genes detected at any of the time-points for each qPCR assay.

### Effects of the intervention on selected 16S rRNA phylotypes

**Figure 1:**
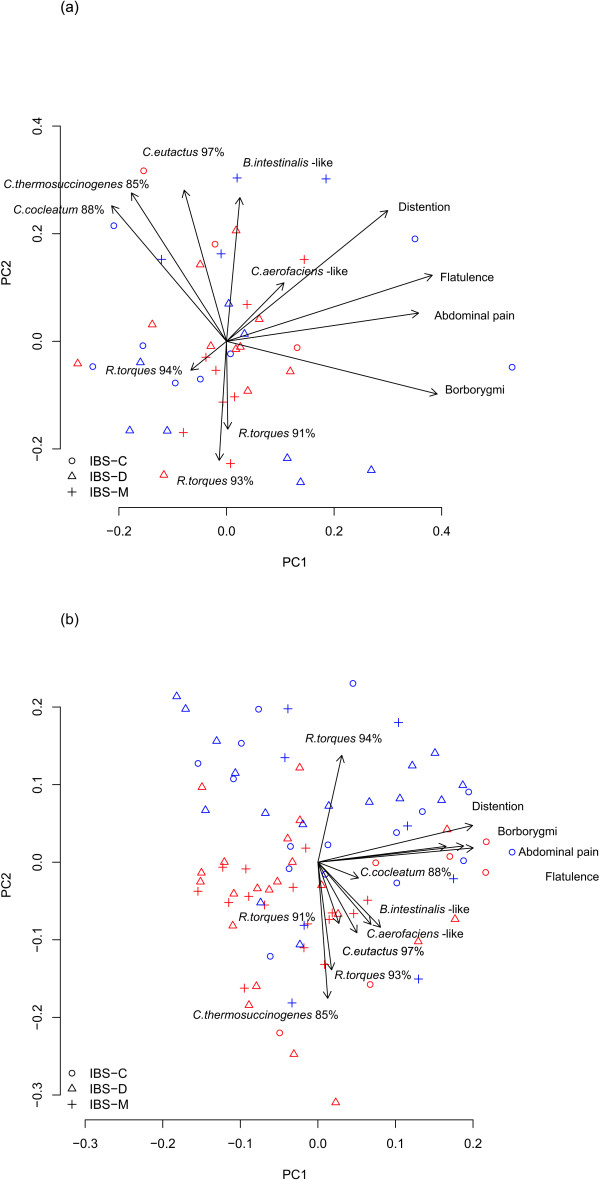
**Principal component analysis for bacterial phylotypes and gastrointestinal symptoms**. Principal component analysis (PCA) for eight bacterial 16S rRNA gene phylotypes and four gastrointestinal symptoms in the (a) before the probiotic intervention (0 months) and (b) during the intervention (combined second and third time-points, 3 and 6 months). Placebo and probiotic groups are denoted in blue and red, respectively. The arrows in the biplot represent the association of the original variables with the samples in the PCA visualization: their length and location are proportional to the variable loadings on the two first principal components. In Figure 1a, the first and second principal components (PC1 and PC2) explain 20.3% and 15.3% of the observed variation, respectively. In Figure 1b, the first and second principal components explain 24.6% and 15.4% of the observed variation, respectively.

In the visualization of the intervention samples with PCA, the placebo and probiotic groups appeared to overlap at the beginning of the study, and no clear associations were seen based on the measured phylotypes or the GI symptoms (Figure 1a). However, in the PCA of samples taken during the consumption of the probiotic combination, the placebo group shifted in the direction of GI symptoms and the probiotic group in the opposite direction (see PC1 in Figure 1a and 1b). The placebo group and the *R. torques *94% phylotype and the probiotic group and the *C. thermosuccinogenes *85% and *R. torques *93% phylotypes pointed in the same directions (see PC2 in Figure 1b).

**Figure 2:**
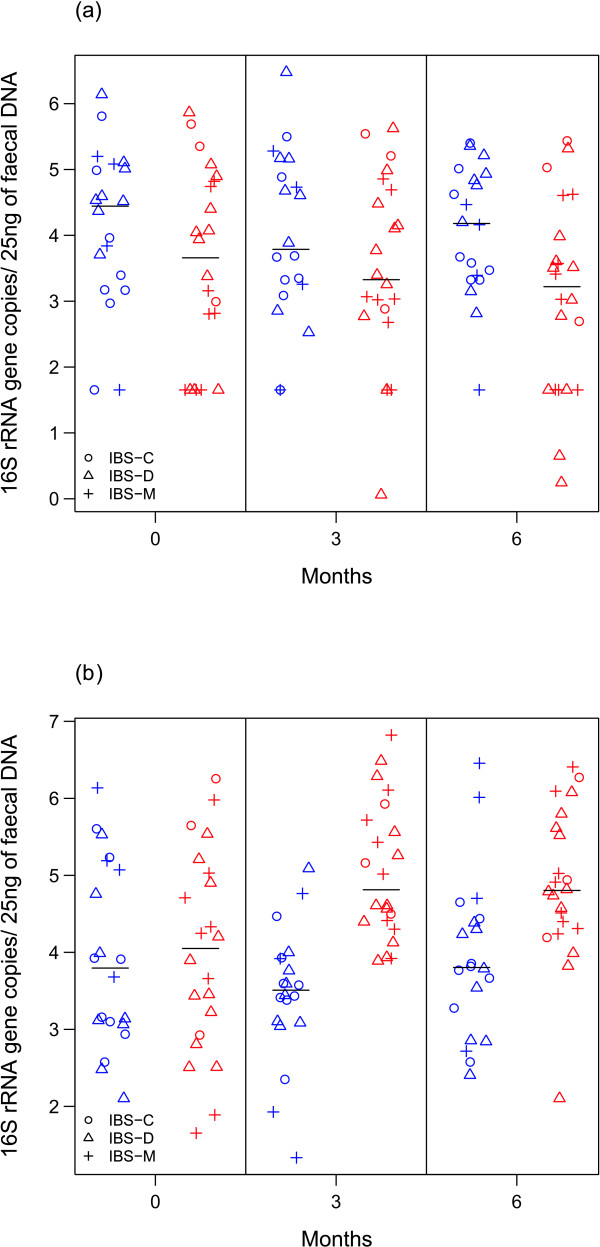
**Quantites of *Ruminococcus torques *94% and *Clostridium thermosuccinogenes *85%**. Stripcharts of (a) *Ruminococcus torques *94% and (b) *Clostridium thermosuccinogenes *85% 16S rRNA gene quantities detected (log**_10_**) with qPCR in the faecal samples of irritable bowel syndrome subjects. Vertical black lines are median values. Placebo and probiotic groups are denoted in blue and red, respectively.

A significant decrease in the amount of *R. torques *94% was observed in the probiotic group as a whole (*P *= 0.02 at time-point 6 months; Table 3 and Figure 2a). Among IBS-D patients, the *R. torques *94% phylotype was significantly more abundant in the placebo group than in the probiotic group at the beginning of the intervention, before consumption of the probiotic supplement (*P *= 0.04 at 0 month; Table 3). The level remained similar for the placebo IBS-D subjects, but decreased among the probiotic-consuming IBS-D subjects, reaching a significant difference relative to the placebo IBS-D patients after 6 months (*P *= 0.01 at 0 month; Table 3). Within the placebo IBS-D subjects, none of the time-points differed from each other significantly, whereas the third time-point in the probiotic group of IBS-D subjects was significantly different from the other two time-points (*P *= 0.01 and 0.02 for comparisons between time-points 3 months vs. 6 months and 0 months vs. 6 months, respectively).

*C. thermosuccinogenes *85% was elevated significantly in the probiotic group as a whole (*P *= 0.00 and *P *= 0.02 at time-points 3 months and 6 months, respectively; Table 3, Figure 2 b) and among IBS-D subjects (*P *= 0.01 at 3 months and *P *= 0.05 at 6 months; Table 3). The effect was stable throughout the intervention among IBS-D subjects (*P *= 0.00 and *P *= 0.04 for comparisons between time-points 0 month vs. 3 months and 0 month vs. 6 months, respectively), and no significant alterations were detected among the placebo IBS-D subjects.

The abundance of *R. torques *93% was higher in the probiotic group during consumption of the probiotic (*P *= 0.00 and *P *= 0.00 at time-points 3 months and 6 months, respectively), but among IBS-D patients the difference disappeared by the end of the study (*P *= 0.00 at 3 months and *P *= 0.50 at 6 months; Table 3). For *R. torques *93%, the detected alterations were due to a decrease in the placebo group.

## Discussion

Our study assessed the effect of a multispecies probiotic supplement on the GI microbiota of IBS patients at a 16S rRNA gene phylotype level in a 6-month placebo-controlled intervention trial [17]. Kajander *et al*. [17] have previously documented that the current intervention significantly reduced the total GI symptom score, mainly due to less borborygmi experienced in the probiotic group. Accordingly, the probiotic group appeared less strongly associated with the monitored IBS-related GI symptoms in the multivariate visualization in this study. The intestinal microbiota during the intervention has subsequently been investigated by applying qPCR targeting bacterial groups and species, but although all supplemented strains were detected, the members of the intestinal microbiota measured were found to remain stable during the intervention, with the exception of *Bifidobacterium *spp., which decreased significantly in the probiotic group [18].

In this study, we applied assays targeting *Bacteroides intestinalis*-like, *Clostridium cocleatum *88%, *C. thermosuccinogenes *85%, *Collinsella aerofaciens*-like, *Coprococcus eutactus *97%, *R. torques *91%, *R. torques *93% and *R. torques *94% phylotypes, which have been shown to diverge between different IBS symptom subtypes and healthy control subjects free of GI symptoms [9,11]. The quantities of these phylotypes together with the IBS-related symptom score were clearly able to differentiate the probiotic-consuming subjects from the placebo group in a PCA of samples taken during consumption of the probiotic combination. Of the bacterial phylotypes, *R. torques *94%, *C. thermosuccinogenes *85% and *R. torques *93% were significantly affected during the intervention.

The *C. thermosuccinogenes *85% phylotype was previously found to be more strongly associated with IBS-M subjects and healthy controls than with patients suffering from IBS-D [11]. The number of bacteria targeted with the *C. thermosuccinogenes *85% assay is increased with multispecies probiotic supplementation. In fact, if per gram of faeces values are calculated, the probiotic intervention seems to lead to higher quantities of the *C. thermosuccinogenes *85% phylotype than previously reported for healthy control subjects devoid of GI symptoms [11] (data not shown).

The bacterial species present by assays targeting phylotypes *R. torques *91%, 93% and 94% may have different metabolic and functional roles in the setting studied, as they were found to behave differently. According to their 16S rRNA gene sequence, these three ruminococcal phylotypes show less than genus level similarity among each other and 91%, 93% and 94% similarity to the species *R. torques*. *Ruminococcus torques *is a mucin-degrading *Clostridium coccoides *group firmicute of the human GI microbiota [23] which has been associated with Crohn's disease [24,25]. The phylotype presented by *R. torques *94% and the target sequence (EMBL: AM275522) of the assay have been associated with IBS-D [11] and Crohn's disease [26], respectively. The closest known bacterial isolate to *R. torques *94% is a fructan-utilizing strain D8 isolated from rat faeces (98% identity with 16S rDNA sequence AY960564) with low-level inulin utilization capability [27]. The phylotype *R. torques *91% has been associated with IBS-D and IBS-M [11] and the phylotype *R. torques *93% has been associated more strongly with healthy control subjects than with IBS-M sufferers [11]. The phylotypes *R. torques *91% and 93% affiliate to strain SSC/2 16S rDNA sequence identity 96% and 99% to AY305320, respectively [28]. Strain SSC/2 is potentially beneficial to health due to its capability to convert lactic acid to butyric acid [29]. However, the mechanisms that link *R. torques *and related phylotypes with an inflamed or irritated intestine remain unknown.

According to the PCA visualization, the placebo group samples appeared to be more strongly associated with *R. torques *94% than the probiotic group samples after the consumption of the probiotic combination had begun. In the assay-specific analysis, a significant decrease was detected by the end of the 6-month intervention in the level of *R. torques *94% among the probiotic group and the IBS-D subjects within the probiotic group. *R. torques *91% did not show alterations due to the consumption of the probiotic supplement, but the abundance of the *R. torques *93% phylotype decreased in the placebo group.

Discrepancies observed between time-points may be due to the effect of the probiotic being prolonged (no effect at 3 months, but effect at 6 months) or being overcome by residents of the GI microbiota (effect at 3 months, but no effect at 6 months). As stress [30] and diet [31] alter the composition of mammalian GI microbiota, a psychological or habitual response to participating in a probiotic intervention could also be reflected in the GI microbiota and be overcome with time. The assay for *R. torques *93%, for instance, also quantifies a lactic acid consuming bacterial strain SSC/2 [29], which could well react to participants being prohibited of using any other probiotic products. A similar effect may apply to *R torques *94%, as it is close to an isolate capable of utilising compounds used in prebiotics [32,33], consumption of which might also alter due to participation in an intervention trial (although prebiotics were not prohibited). Moreover, the phylotypes quantified may represent bacteria in the GI tract with abundances varying over time, especially as IBS patients are known to have an unstable GI microbiota relative to healthy control subjects [7]. Defecation frequencies did not, however, significantly change during the intervention in any of the IBS symptom subtype groups [17]. Nevertheless, independent sample panels still need to be investigated to confirm suspected IBS-related alterations detected in the GI microbiota. Moreover, studies not restricted to certain bacteria or phylotypes are warranted, as are studies going beyond phylogeny, i.e. exploring the metabolism of IBS related GI microbiota.

Our findings support those of Kajander and colleagues [34], who presented a stabilizing effect of multispecies probiotic supplementation (*L. rhamnosus *GG, *L. rhamnosus *Lc705, *P. freudenreichii *ssp. *shermanii *JS and *Bifidobacterium animalis *ssp. Bb12) on the overall GI microbiota of IBS patients during a 5-month intervention. Indeed, the alterations detected here also show a trend towards the quantities previously detected in non-IBS controls free of GI symptoms [11]. The effects of probiotic strains or combinations are unique, and not all probiotic supplementations have favourable clinical effects on IBS symptoms [16]. Certain probiotic strains and multispecies supplements may enhance the expression of mucin components and protect the epithelial layer by adhering to it, thus preventing mucolytic bacteria from digesting the mucus and making the epithelial barrier more vulnerable [35]. Such probiotic strains enhance the barrier function and could alter the quantities of mucolytic bacteria. Studies such as this one are required to improve our knowledge about the mechanisms of actions behind clinically efficient probiotics. This will help us to target the therapy to those patients in the heterogeneous group of IBS sufferers, most likely to respond. Increased knowledge may also provide new insights into the screening of potentially efficient strains.

## Conclusions

Our results indicate that a multispecies probiotic supplement capable of alleviating IBS symptoms affects IBS-associated faecal bacterial phylotypes. Dysbiosis-like changes in the overall GI microbiota and even among specific bacterial phylotypes might have a crucial role in IBS aetiology and pathology, and one potential mechanism underlying the effectiveness of probiotics in IBS may be by affecting these microbes. Although the role of bacteria in IBS aetiology remains uncertain, our methodological approach of using bacterial phylotype-targeting qPCR assays based on IBS-associated clone libraries has revealed potential non-invasive biomarkers, i.e. the *C. thermosuccinogenes *85% and *R. torques*-like phylotypes, for use in IBS associated studies.

## List of abbreviations

IBS-C: Constipation-predominant; IBS-D: Diarrhoea-predominant IBS; GI: gastrointestinal; IBS: irritable bowel syndrome; IBS-M: mixed symptom subtype; PCA: principal component analysis; qPCR: quantitative real-time polymerase chain reaction.

## Competing interests

RK and KKa were employed by Valio Ltd at the time of the study.

## Authors' contributions

AL, LKK and EM coordinated and analysed the qPCR assays. JN and KKu conducted the computational data analyses. KKa planned the clinical trial protocol, recruited the IBS subjects and planned and coordinated the collection of samples. AP and RK coordinated and supervised the study. AL wrote the manuscript, and all authors made corrections to and approved the final manuscript.

## Pre-publication history

The pre-publication history for this paper can be accessed here:

http://www.biomedcentral.com/1471-230X/10/110/prepub
